# The Quantum Environment in Cryptochrome Enhances Light Absorption of FAD

**DOI:** 10.64898/2026.04.24.720615

**Published:** 2026-04-28

**Authors:** Luc Wieners, Martin Garcia

**Affiliations:** 1 Institute of Physics, University of Kassel, Heinrich-Plett-Str. 40, Kassel, 34132, Hesse, Germany.

**Keywords:** cryptochrome, light absorption, quantum mechanics, circadian rhythms

## Abstract

The light absorption of the protein cryptochrome and its chromophore FAD is important for the regulation of circadian rhythms and in some species for sensing magnetic fields. To compute the absorption spectrum of chromophore, typically only a small region is treated quantum-mechanically due the high computational cost of spectroscopic calculations. We present a formalism that allows a quantum-mechanical treatment of not only the chromophore but also the neighbouring amino acids which differ from species to species. This is achieved by using the real-time time-dependent Hartree-Fock method. This method allows extending the quantum domain from typically only a few dozen atoms up to around 1,200 atoms for the largest calculations. The presented framework allows the treatment of neighbouring tryptophan residues or the cofactor molecule MTHF in the same calculation and allows to extract information of which regions absorb light depending on wavelength. The presented results also show that the environment around the chromophore FAD amplifies the light absorption in cryptochrome.

## Introduction

Cryptochromes are proteins which play a main role in sensing light in plants and animals. This is achieved by their chromophore molecule FAD which absorbs blue to ultraviolet light. Light absorption in this ligand leads to a cycle of chemical reactions which causes conformational changes in the protein. The exact changes in the protein and how it signals incoming light to the organism are still an area with ongoing research [1–6].

For these reasons, cryptochrome was identified as an important protein in maintaining circadian rhythms in organisms [7]. Next to acting as a photoreceptor it also plays a role in inhibiting the transcription of DNA. Furthermore, cryptochrome is involved in sensing magnetic fields in many species [8–10]. The sensing is proposed to happen via a radical pair mechanism [11] between FAD and a neighbouring residue which is a phenomenon only observed in cryptochrome.

Consequently, cryptochrome and its ligand FAD has been a target both experimental [12, 13] and theoretical [14–17] studies to gain better understanding of the underlying mechanism of light capture in this protein family. For the theoretical studies, a major challenge is the size of the protein cryptochrome as it consists of around 8,000 atoms, leaving the complete protein out of reach for a fully quantum-mechanical calculation of its absorption. Focussing on the ligand FAD is therefore sensible as it has the central role in the photocycle process. However, the molecule FAD can already be considered quite large for spectroscopic studies since it contains since it contains around 85 atoms, depending on the oxidation state. While one study reports a completely quantum-mechanical treatment of FAD [14], another approach is to investigate only the flavine group consisting of three aromatic rings with quantum mechanics [15, 16] since the absorption of ultraviolet and blue light is known to happen there.

While a reduction of the quantum region is excellent for increasing computational speed or to even make a study computationally feasible, it neglects the environment of the quantum region. Therefore, this approach is commonly coupled with another way of accounting for the environment or the rest of the molecule. A common method is to combine molecular mechanics (MM) [18–21] and quantum mechanics (QM) [22, 23] which is called QM/MM [24].

Simulations of this kind provide the ability to study very large systems and are therefore considered as state-of-the-art in large-scale spectroscopy. The drawback of the QM/MM methodology is that the MM part does not describe the system at a subatomic level and therefore brings limitations. Consequently, a fully quantum-mechanical framework is needed to study environment effects with the inclusion of electronic degrees of freedom.

Here, we achieve this by using the time-dependent Hartree-Fock method (TDHF) with a minimal basis set which allows fast calculations even for very large systems [25]. As an additional point next to presenting calculations with a large-scale quantum region, we also investigated the differences between cryptochrome absorption among species by performing calculations for seven cryptochrome samples for which experimental reference spectra are available. Furthermore, the fine structure of absorption peaks is investigated by computing absorption spectra for structural data of FAD molecules obtained from the protein data bank (PDB) [26].

Finally, we present three examples for absorption spectra calculations of the chromophore FAD in combination with another light-absorbing system, studied in the same system with a quantum-mechanical environment. These three systems are a chain of tryptophan residues involved in the electron transfer process in two different species and the light-collecting cofactor MTHF which supports the light coupling in some cryptochromes. In these systems, 1,100 to 1,300 atoms are treated quantum-mechanically which makes them – to our knowledge – the largest quantum region used for studying light-harvesting proteins.

## Results

### Environment creation

**Figure 1:**
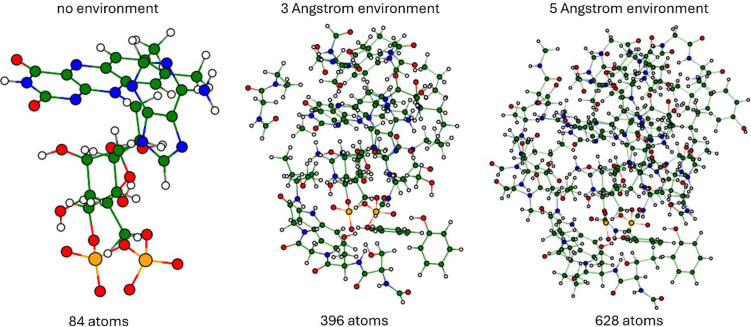
Atomic environments of FAD in cryptochrome. The different environments used for computing the absorption spectrum of cryptochrome are shown: no environment, three and five Angstrom environment. The corresponding atomic configurations, which include additional atoms to ensure sensible boundaries, are depicted for one of the seven systems (PDB 9LPG [27, 28]).

The first two goals in this study are the investigation of the effects of including environment in the quantum-mechanical region and of studying the difference in absorption between species compared to experimental results. A starting point for all calculations is always the chromophore in the form of the molecule FAD. For investigating environment-based effects, we consider different configurations of the environment around the ligand: one configuration with no environment, one with an environment of three and one with five Angstrom (see Figure 1). The procedure of calculating these environments is described in the Methods section.

### Experimental data for cryptochrome in animals and plants

For the procedure described above, the starting point is a structure file containing coordinates of the atoms in cryptochrome. In this part of the study, we focus on a comparison with preexisting experimental results from different groups [27–39]. For this purpose, we chose structures where an experimentally obtained absorption spectrum is available. The allocation of these configurations was done in the following ways. First, a search through all cryptochrome structures deposited on the RCSB protein data bank (PDB) [26] was performed. Then, those ones where both FAD is bound to cryptochrome and where an experimental spectrum of the same molecule was published in the paper corresponding to the PDB entry were selected. Additionally, samples with further functional complexes involved which changed the experimental absorption spectrum were avoided as well. In total, this leads to seven cryptochrome structure files with suitable experimental reference data for this study. The corresponding PDB entries are 9LPG [27, 28], 6K8I [29, 30], 6K8K [29, 31], 6X24 [32, 33], 6PU0 [34, 35], 6WTB [36, 37] and 8P4X [38, 39] and the respective species names are *Zea Mays*, *Arabidopsis thaliana*, *Arabidopsis thaliana*, *Arabidopsis thaliana*, *Columba livia*, *Drosophila melanogaster* and *Platynereis dumerilii*. This means that the dataset contains cryptochromes from maize, thale cress, rock pigeon, fruit fly and clam worm and consequently consist of both plants and animals.

### Computational method

In the absorption spectra calculations, we use the real-time time-dependent Hartree-Fock (RT-TDHF) method. This choice is motivated by a number of reasons. First, real-time methods scale at a computational cost of *Ο*(*n*^3^) while linear-response methods can scale up to *Ο*(*n*^6^) if all eigenvalues are considered. Secondly, a central part in the computational complexity of real-time time-dependent Hartree-Fock or density functional theory is the construction of the Hamiltonian which needs to be done at every time step during the time propagation. For systems with a few hundred atoms this step is commonly much more time-consuming than the eigenvalue calculation and therefore is considered a major bottleneck in computation time since the Hamiltonian matrix is computed thousands of times during a RT-TDHF or RT-TDDFT calculation [40]. The Hartree-Fock method allows a fast calculation of the Hamiltonian, especially the electron-electron interaction, since the integrals can be precomputed and stored which allows a fast assembly of the electron-electron tensor at each time step. This enables calculations with over 1,000 atoms.

A final advantage of RT-TDHF is that the Hartree-Fock method scales favourable for biophysical systems which have a lower density than for example systems in solid state physics. This leads to less integrals in total and a fast computation of the Coulomb and exchange interaction in the aforementioned building of the Hamiltonian. Our code NIMBLE [25] is focused on fast Hartree-Fock calculations and filters out insignificant density interactions for further speedup. A detailed description of the used algorithms is given in the corresponding reference.

It has to be mentioned that this approach loses accuracy due the neglection of electronic correlation inherent to the Hartree-Fock method. Furthermore, we use the minimal basis (STO-3G) to maximize computational speed which introduces further errors. However, especially for the case of theoretical spectroscopy, a large part of these errors can be compensated by systematically comparison with reference data and reference calculations [41, 42]. The approaches to recognize, correct and control errors in this formalism are described in the following sections.

These considerations come at the advantage of the mentioned ability to study larger systems previously out of reach as well as reasonable run times, making calculations accessible to smaller computation clusters or even personal computers, therefore saving time and energy. For the quantum-mechanical time propagation, a protocol similar to [40] is used, utilizing the predictor-corrector time integration method. The propagation is done over 10,000 steps in total with a time step of 0.25 atomic units of time which leads to a total propagation time of 60.5 fs. The relatively long computation time ensures an accurate capture of dynamics at larger wavelengths and also allows a detailed resolution of the absorption peaks needed for quantitative comparisons with experiments. The Fourier transform uses an attenuation factor of 0.015 atomic units unless specified otherwise.

### Computational and experimental absorption spectra

To begin with, we want to compare the computed spectra of the seven samples with their respective experimental spectra and then investigate the effects of different environment sizes. For a comparison with experimental spectra, it is common practice to shift the theoretical spectra by a constant wavelength to account for systematic errors [14, 16, 41, 42]. Here, this calibration is done with the spectra obtained using the largest environment as this calculation should represent the biological system the best. A shift of 140 nm was found to be optimal. Note that we expect high shifts due to the use of the Hartree-Fock methods which systematically overestimates HOMO-LUMO gaps leading to absorption at lower wavelengths. In Table 1, the positions of both peaks are given for the experimental and theoretical spectra with the discussed shift used for the latter. Here, an attenuation factor of 0.005 was used in the Fourier transform for a better resolution of the peaks.

If a smaller attenuation factor is used in the Fourier transform, we find that the major absorption peak of FAD at around 370 nm consists of two peaks in the theoretical spectrum. These are however not related to the two peaks in the experimental spectrum. Instead, one of the absorption peaks is caused by the adenine at the adenosine diphosphate (ADP) end of the FAD molecule. In the calculations, these two peaks overlap since the adenine, which absorbs at a lower wavelength than the flavine, would need a different shift as the error of its absorption peak is smaller than for the flavine group.

This stresses the importance of reference calculations when using the RT-TDHF framework to adequately interpret absorption peaks. Consequently, one has to keep in mind that the first absorption peak has a strength which is about twice as high as expected due to the overlapping absorption of the adenine. If this is accounted for, both relevant peaks have a similar intensity as it is the case for the experimental spectra.

**Figure 2:**
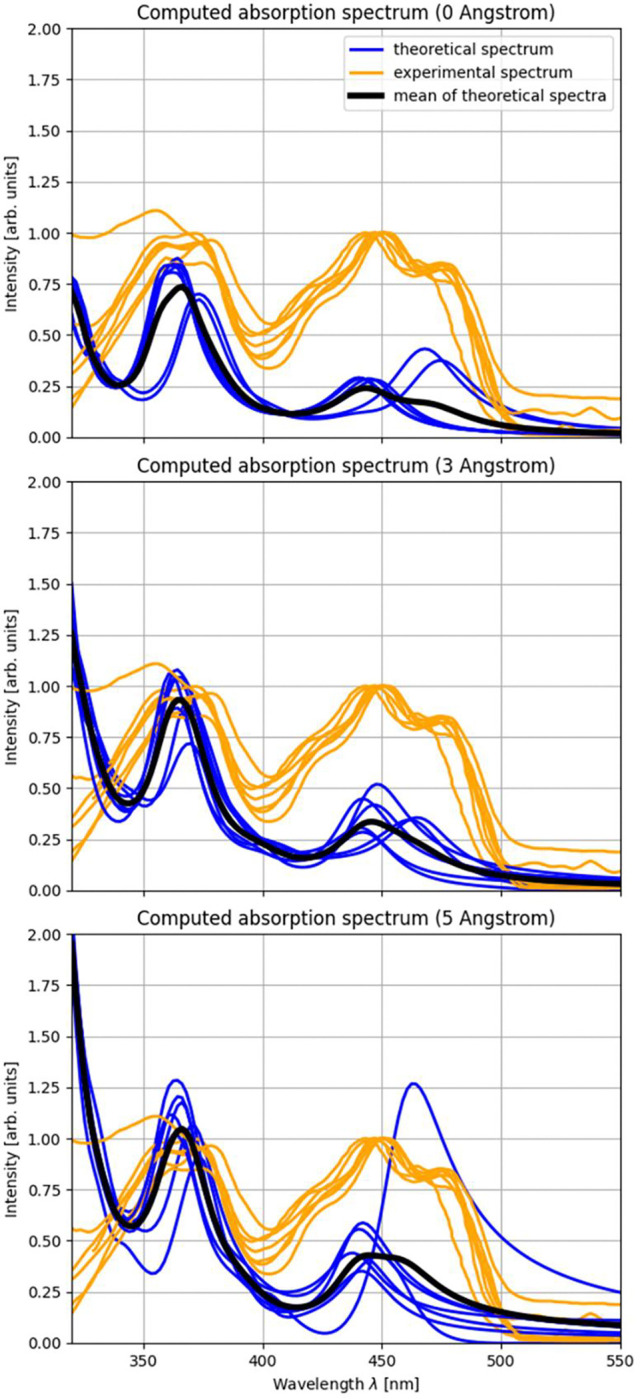
Absorption spectra in different environments. Comparison between experimental and theoretically obtained absorption spectra. The experimental spectra from the seven studied samples are shown in orange and the theoretical samples in blue, with the mean of the theoretical samples visualized as the black line. The top, middle and bottom panel show a comparison for environment sizes of 0, 3 and 5 Angstrom, respectively.

Now, we investigate the difference in the absorption spectra for different environment sizes. This is shown in Figure 2 where the experimental and theoretical spectra are shown for all seven samples for the cases of no environment, 3 Angstrom and 5 Angstrom environments. It can be observed that the peak positions of the computed spectra agree well with the experimental peak positions in all environment configurations and not just for the 5 Angstrom environment which was used for the calibration. Consequently, for the case of FAD embedded in cryptochrome, the environment appears to not influence the positions of the absorption peaks of the chromophore in a significant way. This result is in good agreement with experimental data since the experimental cryptochrome absorption spectra used in this study agree strongly with the spectrum of isolated FAD [43].

**Figure 3:**
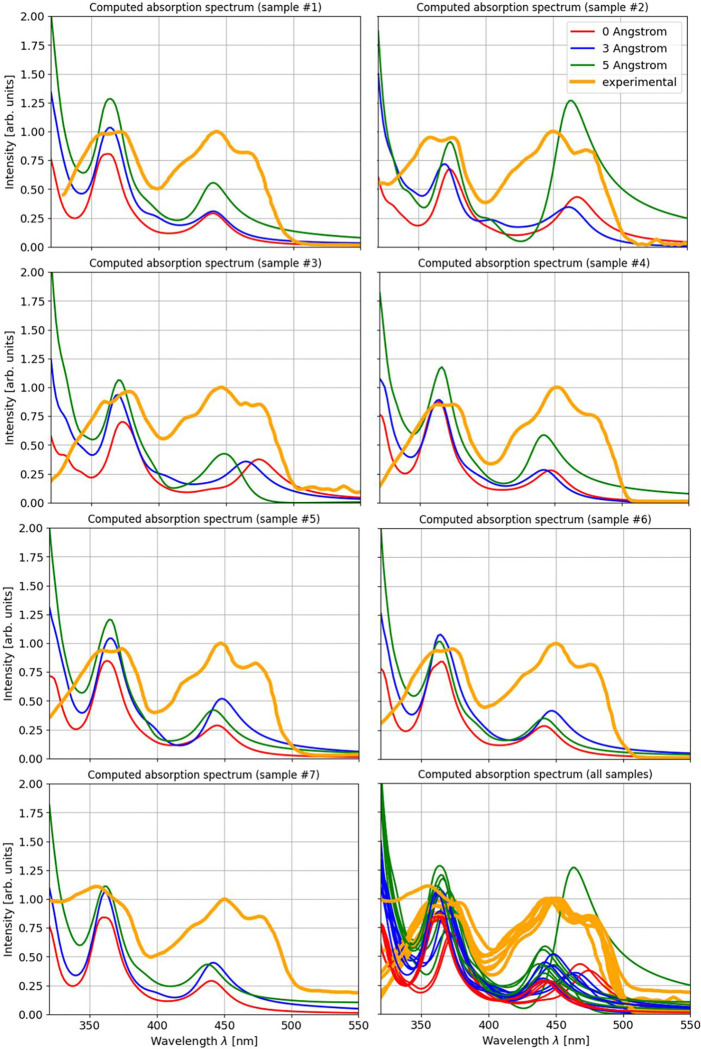
Absorption spectra for different cryptochrome samples. Comparison of spectra for different environment sizes and different samples. The spectra are shown for all 7 samples individually with the experimental and the three theoretical spectra displayed. In the bottom right panel, all spectra for all samples are visualized together.

Next to the absorption peak positions, the intensity of the absorption can be investigated over different environment sizes. In Figure 2 it can be seen that the theoretical spectra (in blue) show an increase in intensity for an increasing environment size, both for the step up from 0 to 3 Angstrom and from 3 to 5 Angstrom. One sample shows a very strong absorption at the second absorption peak of FAD for the 5 Angstrom environment. The intensity differences are also shown in more detail in Figure 3 where the three environment configurations are shown in comparison with the respective experimental spectrum for each of the samples individually. An increase of the intensity of the absorption between 0 and 5 Angstrom environments can be seen for all systems at both major absorption peaks. In the bottom right panel of Figure 3, all absorption curves are visualized together to illustrate this trend.

### Details of the FAD absorption spectrum

Another observation relevant for studying the absorption profile of FAD in detail is the fine structure of the absorption peaks. The two major absorption peaks of FAD are observed around 370 nm and 450 nm. However, the first peak is a doble peak with absorption maxima at 355 nm and 375 nm, with the absorption at 375 nm being slightly higher than the absorption at 355 nm. Furthermore, the second peak consists of absorption at three different wavelengths. It has its overall maximum at 445 nm and a second maximum at 475 nm as well as a weaker peak at 425 nm.

These fine structure features are however not visible in computational studies – for both structures (the double and the triple peak) there is always only one maximum reported (note that one peak of the double peaks in this study is caused by the ADP end of FAD). Therefore, we performed calculations for a larger set of existing structures of FAD molecules bound into cryptochrome. This list and its PDB accession codes are discussed in the methods section. In total it contains 28 structures for which the light absorption was computed in the same way as for the list of seven samples discussed above. In these calculations, no environment was used to focus on the absorption spectrum of FAD itself.

**Figure 4:**
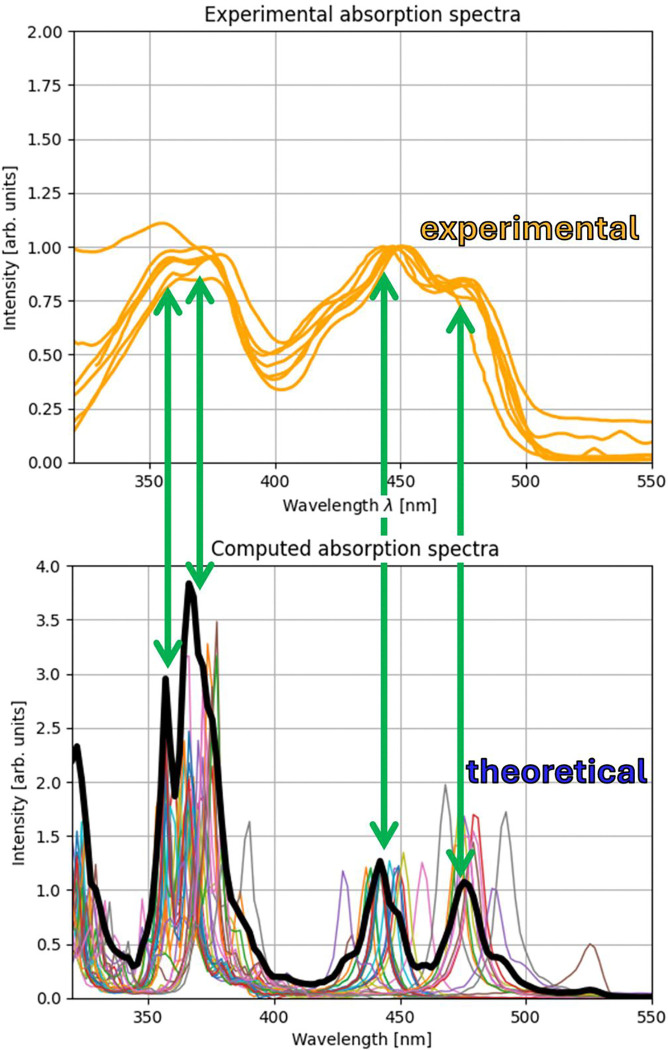
Details of the FAD absorption spectrum. Investigation of 28 FAD molecule structures bound to cryptochrome from the Protein Data Bank (PDB) [26] and comparison with experimental spectra. Top: Seven experimental absorption spectra from FAD in cryptochrome (see previous sections). Bottom: The individual spectra are shown as coloured lines and the average is shown as a black line. For visualization purposes, the average is multiplied by a factor of four. The green arrows connect the absorption peaks from the calculation with the peaks from the experiment. For a sharper visualization of the absorption peaks, an attenuation factor of 0.003 was used in the Fourier transform for these calculations.

The results of these calculations are depicted in Figure 4 where all individual spectra and a mean spectrum of the FAD molecules is shown and compared to experimental spectra. Here, we observe a grouping of the absorption peaks at two different wavelengths for both of the two major absorption peaks of FAD. This results in both of the absorption peaks being split into two when the mean of all absorption spectra is computed.

Especially for the absorption peak at 450 nm, the splitting can be observed clearly in Figure 4 as well as in Figure 2 (most prominently in the top panel) and in Figure 3 in the bottom right panel. This leads to the conclusion that different FAD structures lead to slightly different absorption peaks which has to be caused by the conformation of the FAD molecule since not environment is considered in the calculations presented in Figure 4.

### Systems with multiple absorbing molecules

Finally, we want to test the performance of the presented framework for larger systems in cryptochrome, exceeding system sizes of 1,000 atoms. For this purpose, we chose three systems where additional structures near the chromophore molecule FAD got investigated. First, we studied four tryptophan residues (the so-called “tryptophan tetrad”) [44–46] which are relevant for the electron transport pathway mechanism in the FAD photocycle and also show absorption at lower wavelengths. This was done for two species: *Drosophila melanogaster* (fruit fly) as a model organism and *Columba livia* (rock pigeon) as an organism that utilizes FAD and the tryptophan tetrad for magnetoreception. The PDB accession codes are 4GU5 [47, 48] and 6PU0 [34, 35], respectively.

Second, we investigated a cryptochrome with two ligands, FAD and MTHF from *Arabidopsis thaliana* (thale cress), PDB 2J4D [49, 50]. Here, MTHF is considered to serve as a light-collecting ligand to support the photocycle of FAD. The three considered systems have sizes of 1227, 1291 and 1145 atoms, respectively.

For the tryptophan residues it has to be considered that the shift of the absorption profile will be different from the scaling factor in FAD. Therefore, the shift for tryptophan was computed individually by calculating the absorption spectrum of a single tryptophan molecule. This results in a shift of 50 nm. Due to the shift of FAD being 140 nm, the absorption maxima of FAD (375 nm) and tryptophan (280 nm) can be found close to each other, similar to the absorption maxima of the flavine group and the adenine coinciding in the absorption spectrum of FAD.

**Figure 5:**
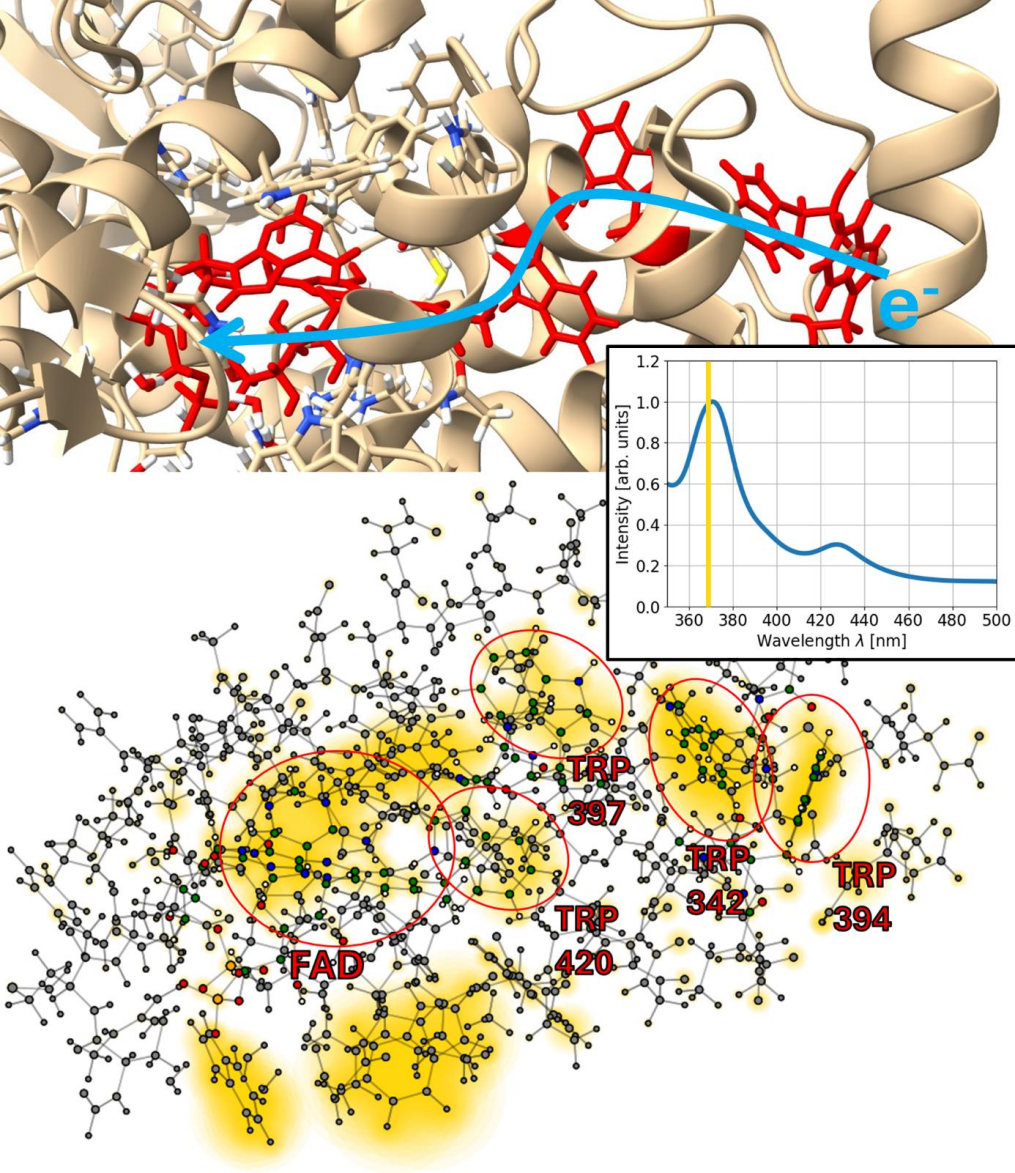
Absorption of the tryptophan tetrad in *Drosophila melanogaster* (fruit fly). Top: Visualization of the investigated system and its environment using ChimeraX [52–54]. The chromophore FAD and the four relevant tryptophan residues (W342, W394, W397, W420) involved in the electron transport are coloured in red with the electron transport being indicated by the blue arrow. Bottom: The investigated system at an atomistic resolution. The core molecule and residues are marked in red with their atoms shown in colour (all other atoms in grey). The absolute values of the Fourier transform of the dipole moments of all atoms are shown as yellow circles with the circle size corresponding to the respective value. They are displayed for a wavelength of 368 nm and indicate regions in which absorption may happen. The computed absorption spectrum is shown as an inset.

**Figure 6:**
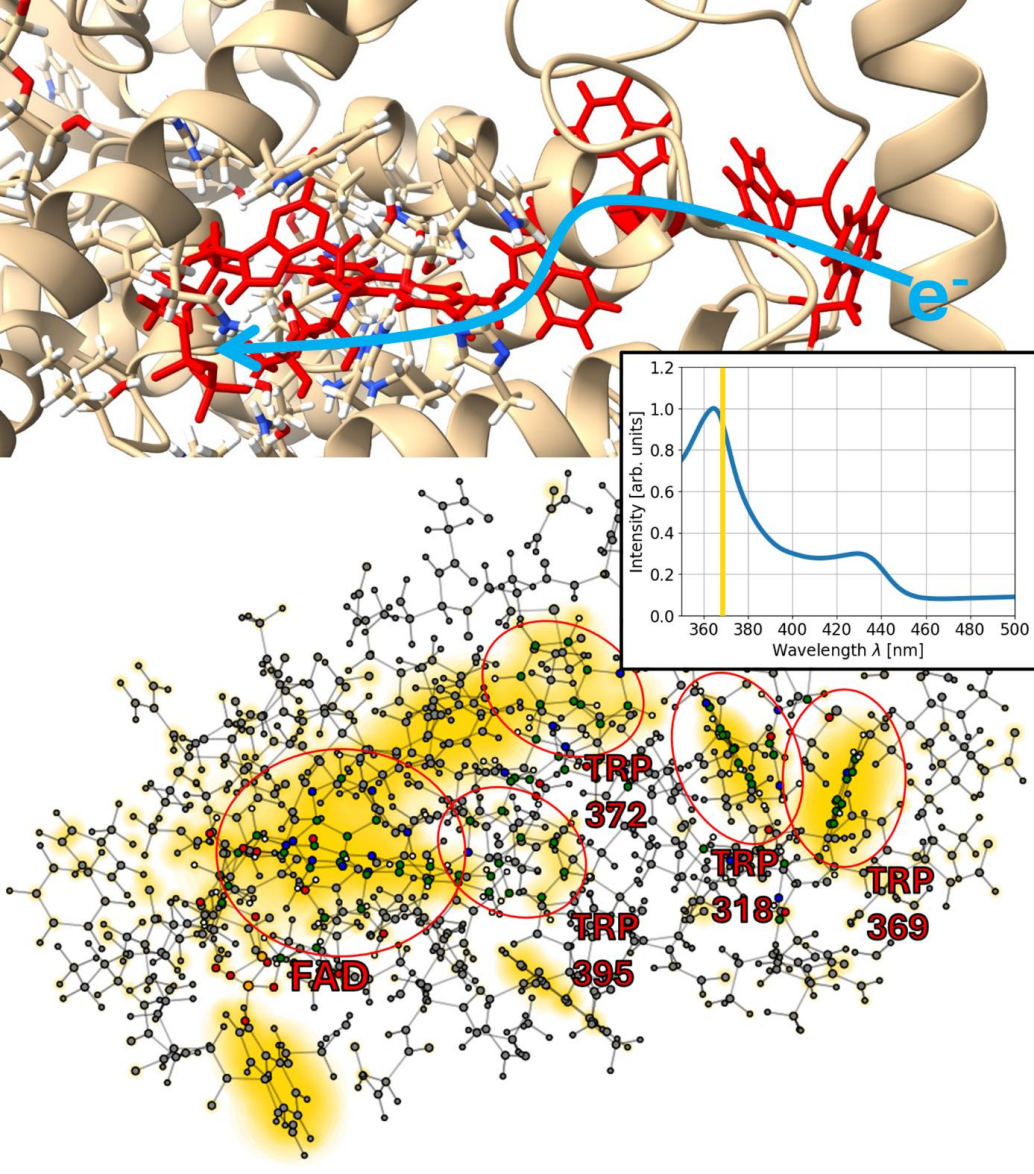
Absorption of the tryptophan tetrad in *Columbia livia* (rock pigeon). Top: Visualization of the investigated system and its environment using ChimeraX [52–54]. The chromophore FAD and the four relevant tryptophan residues (W318, W369, W372, W395) are highlighted. Bottom: A visualization of the absorption regions at 368 nm in the same way as in Figure 5. The computed absorption spectrum is shown as an inset.

**Figure 7:**
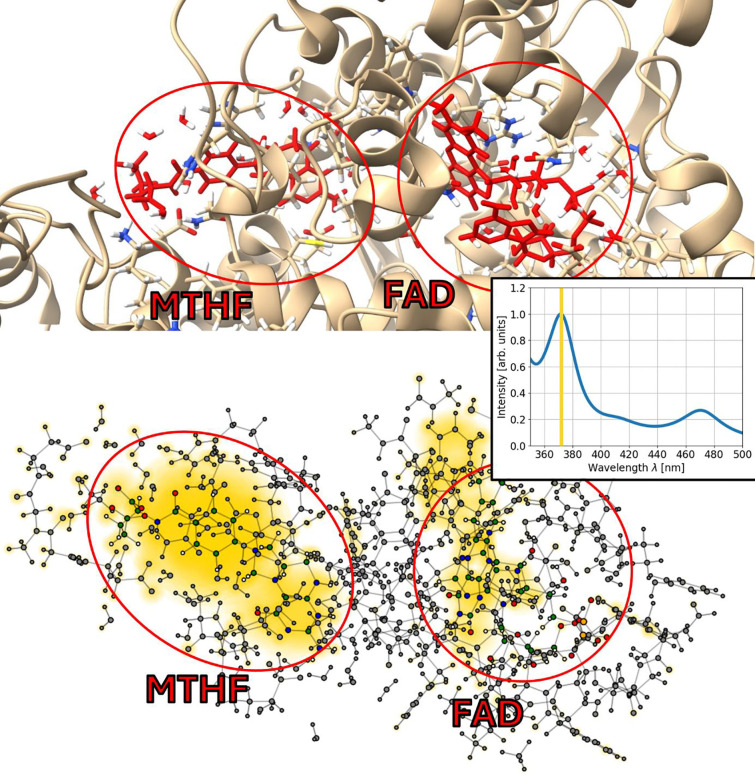
Absorption of MTHF and FAD. The absorption of the cofactor MTHF in cryptochrome is investigated in combination with the absorption of the chromophore FAD. Top: Visualization of the structure using ChimeraX [52–54] with the two ligands highlighted and circled in red. Bottom: Absorption at 372 nm is displayed in the same way as in the previous two Figures. The computed absorption spectrum is shown as an inset.

To visualize where the absorption takes places, one can compute the Fourier transform not only for the total dipole molecule of the system but also for the dipole moments of the individual atoms [51]. This has been done for the three systems described above and allows to investigate where and at which strength the absorption takes place. In Figures 5, 6 and 7 this is shown for the three studied molecules.

In total, we observe absorption in FAD as well as in the tryptophan residues and MTHF. This is expected, since tryptophan is the amino acid with the strongest absorption and MTHF is known to absorb at around 370 nm. [49, 55, 56]

Consequently, the highest absorption peak in Figure 7 is attributed to FAD and MTHF, where MTHF shows stronger absorption than FAD, according to the visualization in Figure 7. For the systems with FAD and the tryptophan tetrad, the highest absorption is also found at 370 nm, this time as a combination of the absorption of FAD and the tryptophan residues (see Figures 5 and 6). Due to the absorption shift of tryptophan being 90 nm smaller than the shift of FAD, the absorption maximum corresponds to 280 nm for the tryptophan residues which is in agreement with the absorption peak position of isolated tryptophan.

For the systems with the tryptophan triad, the high absorption could be relevant for the electron transport as well since high absorption also corresponds to high polarizabilities.

The MTHF-FAD system shows a stronger absorption for MTHF than for FAD which corresponds well to the role of MTHF as the light-harvesting cofactor. It also matches experimental data of cryptochrome absorption spectra with FAD and MTHF where the absorption peak of MTHF dominates [49, 55].

## Discussion

The presented framework for quantum-mechanical absorption spectra calculations allows the study of biomolecules bound into proteins with a fully quantum-mechanical treatment of the environment in close proximity.

Certainly, a downside of the described methodology is the higher error associated with the RT-TDHF calculations in a minimal basis. Therefore, the results often cannot be used without further investigation regarding the origins of the absorption peaks. Despite having multiple absorption overlaps at around 370 nm in this study, we found that using experimental reference spectra and reference calculations of isolated molecules, these errors can be controlled reliably. We hope that the presented protocol can be used to study light absorption processes with a quantum-mechanical environment in many more systems. The option of an explicit quantum-mechanical treatment of surroundings is promising to be of interest for a wide variety of absorption spectra calculations. This is especially the case if the environment is known to play an important role for the absorption spectrum or if parts of the environment are absorbing as well at a different wavelength. Furthermore, the presented method can also be used to study the effect of solvents for larger structures with the QM region including the solvent.

In addition to this, larger quantum regions allow the treatment of multiple absorbing molecules and their environment in a single system, as demonstrated for MTHF and a chain of tryptophan residues in this study. A first step into studying the tryptophan residues in the electron transport chain could also be interesting for investigating magnetoreception since the chain of four tryptophan residues is postulated to play an important role in this topic [57, 58].

## Conclusion

We presented calculations of the light-sensing protein cryptochrome and its cofactor FAD while treating the close-range environment around the ligand fully quantum-mechanically. This presented method of QM environments for absorption spectra calculations was used to study seven structures of FAD bound to cryptochrome from multiple plants and animals. We observed that the region around the chromophore in cryptochrome leads to an amplification of the light absorption which in the protein is higher than in isolated FAD. Furthermore, we studied systems with multiple light-absorbing molecules, exceeding 1,000 atoms in total.

By doing so, we provided large-scale and systematic investigations regarding the light absorption of the chromophore FAD bound to cryptochrome regarding different environments, different species and in the presence of other light-absorbing molecules. Additionally, we provide a framework for studying absorption spectra of molecules with a quantum-mechanical treatment of the surrounding protein.

## Methods

### General programme information

All absorption spectra calculations were performed using the real-time Hartree-Fock method implemented in our software NIMBLE. For the code and its detailed parametrization, we refer the interested reader to reference [25]. The STO-3G basis [59] is used and density-density interactions are truncated at a threshold of 10^−6^. Coulomb interactions are not truncated in the calculations.

Matrix operations are done with pytorch [60] while the computation of the electronic exchange matrix is done with a custom python routine which is just-in-time compiled and parallelized using the library numba [61], plots were done with the matplotlib library [62]. The RTHF calculations can also be parallelized among 3 compute nodes which calculate the responses for the electric field in x-, y- and z-direction individually.

Most calculations were performed on compute nodes with 48 cores and took between a few hours to three days. For larger structures, it is necessary to have random access memory (RAM) in the range of 100–200 Gigabytes to ensure that the precomputed electron repulsion integrals, of which there are up to 7 billion for the largest calculation, can be stored. The time evolution of the dipole moments of the complete structure as well as for the individual basis functions are stored after the calculation is completed.

Coordinates and elements as well as the charge of the structure were precomputed and then read in again during the RTHF calculations. The procedure for precomputing these inputs is described in the following paragraph.

### Preparation of atomic coordinates

For the investigation of the differences between the three environment types used in this study (no environment, 3 Angstrom and 5 Angstrom), it is necessary to prepare the corresponding set of atomic coordinates and elements to perform the RTHF calculation. This is done by first identifying all atoms which are have a distance to any of the atoms in the ligand which is less or equal than the given threshold (i.e. 3 or 5 Angstrom). Since only including these atoms would cut bonds on a non-physical way, additional atoms have to be added. This is done in a way so that the cutting of bonds changes the properties of the system as little as possible. For this reason, only single bonds between two carbon atoms are cut. The bond character is determined by computing the distance between the two atoms. Since the coordinates in this study are always taken from PDB files, there is very little deviation in the bond distances of bonds with the same character which makes this a reliable method for bond character determination. If a bond is being cut, the atom outside the added environment is replaced with a hydrogen atom with the bond distance chosen as the distance of a carbon-hydrogen bond.

In addition to preparing the atomic coordinates for the calculations with a protein environment, it is also necessary determine the charge of the system. This is done by testing different charges in the form of adding or subtracting electrons from the system. For each of these tests, a few density matrix propagation steps are performed in the RTHF formalism and the charge of the FAD molecule is computed using Mulliken population analysis. The total charge is then selected among these test runs based on numerical stability and the charge of FAD where the latter should be equal to 408 (or in practice between 407.5 and 408). Note that 408 is two more than the sum of the atomic numbers of all elements in FAD. This is necessary due to the two phosphate groups in FAD which contain a negative charge each.

Consequently, the first criterion for choosing the correct electronic charge was the computed charge of FAD. In cases where multiple total charges lead to a suitable charge of FAD, the numerically stable case was chosen which was unique for all samples. The numerical stability was evaluated by computing the trace of the square of the density matrix which should correspond to the number of electrons in the system (except for a prefactor due to double occupations of orbitals). This is done routinely during RTHF calculations in NIMBLE to ensure that the time propagation runs stable – instable states are then identified due to a strong drift in the trace. The drift in the trace of the square of the density matrix can also be used to investigate the numerical loss or gain of electrons during the time propagation. The largest observed value in this study was found for the system containing FAD and the tryptophan tetrad for *Drosophila melanogaster* with a 5 Angstrom environment; here a deviation of 2.8 · 10^−8^ electrons/electron was observed after the completed time propagation over 60 fs, a value well within the scope of other quantum-mechanical time propagations [63].

The charges used in the calculations in this work are listed below, they are given in elementary charges i.e. 2 corresponds to removing 2 electrons.

- FAD without environment: −2 for all samples.
- CRY-FAD with 3 Angstrom environment: −3, −2, −2, −2, 1, 0, −1, for the seven samples in the order in which they are introduced in the main text.
- CRY-FAD with 5 Angstrom environment: −4, −3, −3, −3, −1, 0, 0, for the seven samples in the order in which they are introduced in the main text.
- TRP tetrad *Drosophila melanogaster*: 1
- TRP tetrad *Columba livia*: 0
- FAD-MTHF system: −6

For the study of the absorption peak positions of FAD without environment, 5 samples (sample numbers 3, 7, 8, 13 and 27 according to the list in the following section) were only numerically stable for charges other than −2. These samples were therefore not used since the focus was to simulate FAD absorption spectra at standard conditions.

Especially for the large-scale systems, the dipole moments were found to have a slight shift after the initial electric field pulse. This was corrected by subtracting the mean of the last 9,000 time steps of the dipole moment evolution for each coordinate before taking the Fourier transform.

Since the atomic coordinates are obtained from PDB files, only heavy atoms have explicit coordinates. The hydrogen atoms have to be added afterwards which was done using ChimeraX [52–54]. The hydrogen arrangement of the flavine group computed by ChimeraX does not match the structure of FAD in its oxidized form which is the molecule investigated in this study. For this reason, one hydrogen atom was deleted from the structures from ChimeraX to achieve the intended state.

### List of files for the investigation of FAD absorption details

For the comparison with experimental data and to investigate the details in the absorption profile of FAD, this study uses structures from the PDB [26]. The structures were obtained by searching for structures with the UniProt molecule names “Cryptochrome-1”, “Cryptochrome-2”, “Cryptochrome2”, “Cryptochrome DASH”, “Cryptochrome DASH, chloroplastic/mitochondrial”, “Cryptochrome/photolyase family protein” which are all listed molecule names that contain the word “cryptochrome”. Out of the PDB entries listed with these molecule names, only those with FAD as a ligand were selected. In total, this leads to 33 structures (as of late 2025).

In the following, a list of these 33 structures [27–39, 47–50, 64–100] is provided: PDB accession code, species name and classification of the relevant molecule according to the PDB entry are given.

1. 6LZ3, *Zea Mays*, Cryptochrome2
2. 9LPG, *Zea Mays*, Cryptochrome2
3. 2IJG, *Arabidopsis thaliana*, Cryptochrome DASH, chloroplast/mitochondrial
4. 2J4D, *Arabidopsis thaliana*, Cryptochrome DASH
5. 2VTB, *Arabidopsis thaliana*, Cryptochrome DASH
6. 7YKN, *Vibrio cholerae*, Cryptochrome/photolyase family protein
7. 8A1H, *Vibrio cholerae*, 6–4 photolyase (FeS-BCP, CryPro)
8. 9HNK, *Caulobacter vibrioides*, Cryptochrome/photolyase family protein
9. 9HNL, *Caulobacter vibrioides*, Cryptochrome/photolyase family protein
10. 9HNM, *Caulobacter vibrioides*, Cryptochrome/photolyase family protein
11. 9HNN, *Caulobacter vibrioides*, Cryptochrome/photolyase family protein
12. 9HNO, *Caulobacter vibrioides*, Cryptochrome/photolyase family protein
13. 9Q8F, *Caulobacter vibrioides*, Cryptochrome/photolyase family protein
14. 1NP7, *Synechocystis sp. PCC 6803*, DNA photolyase
15. 4I6G, *Mus musculus*, Cryptochrome-2
16. 6K8I, *Arabidopsis thaliana*, Cryptochrome-2
17. 6K8K, *Arabidopsis thaliana*, Cryptochrome-2
18. 6M79, *Arabidopsis thaliana*, Cryptochrome-2
19. 6X24, *Arabidopsis thaliana*, Cryptochrome-2
20. 7X0X, *Arabidopsis*, Cryptochrome-2
21. 7X0Y, *Arabidopsis thaliana*, Cryptochrome-2
22. 1U3C, *Arabidopsis thaliana*, Cryptochrome 1 apoprotein
23. 1U3D, *Arabidopsis thaliana*, Cryptochrome 1 apoprotein
24. 4GU5, *Drosophila melanogaster*, Cryptochrome-1
25. 4JZY, *Drosophila melanogaster*, Cryptochrome-1
26. 4K03, *Drosophila melanogaster*, Cryptochrome-1
27. 6LZ7, *Zea mays*, Cryptochrome-1
28. 6PTZ, *Columba livia*, Cryptochrome-1
29. 6PU0, *Columba livia*, Cryptochrome-1
30. 6WTB, *Drosophila melanogaster*, Cryptochrome-1
31. 7UD0, *Drosophila melanogaster*, Cryptochrome-1
32. 8DD7, *Drosophila melanogaster/Homo sapiens*, Methylated-DNA--protein-cysteine methyltransferase, Cryptochrome-1 fusion
33. 8P4X, *Platynereis dumerilii*, Putative light-receptive cryptochrome (Fragment)

## Figures and Tables

**Table 1:** Peak positions of experimental and theoretical FAD absorption spectra. A shift of 140 nm is employed for the theoretical spectra (computed in a 5 Angstrom environment) and absolute and relative errors under this shift are given with respect to the experimental values of the same sample. All values are given in nm, except for the relative errors.

Sample	Peak 1 exp.	Peak 1 theo.	Peak 2 exp.	Peak 2 theo.	Abs. error peak 1	Abs. error peak 2	% error peak 1	% error peak 2
1	365	364	443	439	1	4	0.3	0.9
2	367	374	449	460	7	11	1.9	2.4
3	369	373	447	456	4	9	1.1	2.0
4	369	367	451	440	8	11	2.2	2.4
5	366	364	447	441	2	6	0.5	1.3
6	368	365	451	442	3	9	0.8	2.0
7	365	363	451	438	2	13	0.5	2.9
**average**	**367**	**367**	**448**	**445**	**4**	**9**	**1.0**	**2.0**

## References

[1] Giovani B., Byrdin M., Ahmad M., & Brettel K. (2003). Light-induced electron transfer in a cryptochrome blue-light photoreceptor. Nature Structural & Molecular Biology, 10(6), 489–490. 10.1038/nsb933 12730688

[2] Lin C., & Shalitin D. (2003). Cryptochrome structure and signal transduction. Annual review of plant biology, 54(1), 469–496. 10.1146/annurev.arplant.54.110901.160901 14503000

[3] Ozturk N., Selby C. P., Annayev Y., Zhong D., & Sancar A. (2011). Reaction mechanism of Drosophila cryptochrome. Proceedings of the National Academy of Sciences, 108(2), 516–521. 10.1073/pnas.1017093108 PMC3021015 21187431

[4] Berntsson O., Rodriguez R., Henry L., Panman M. R., Hughes A. J., Einholz C., ... & Westenhoff S. (2019). Photoactivation of Drosophila melanogaster cryptochrome through sequential conformational transitions. Science Advances, 5(7), eaaw1531. 31328161 10.1126/sciadv.aaw1531 PMC6636987

[5] Kondoh M., Shiraishi C., Müller P., Ahmad M., Hitomi K., Getzoff E. D., & Terazima M. (2011). Light-induced conformational changes in full-length Arabidopsis thaliana cryptochrome. Journal of molecular biology, 413(1), 128–137. 21875594 10.1016/j.jmb.2011.08.031 PMC4451184

[6] Li P., Cheng H., Kumar V., Lupala C. S., Li X., Shi Y., ... & Tan Y. W. (2022). Direct experimental observation of blue-light-induced conformational change and intermolecular interactions of cryptochrome. Communications biology, 5(1), 1103. 36257983 10.1038/s42003-022-04054-9 PMC9579160

[7] Cashmore A. R. (2003). Cryptochromes: enabling plants and animals to determine circadian time. Cell, 114(5), 537–543. 13678578

[8] Xu J., Jarocha L. E., Zollitsch T., Konowalczyk M., Henbest K. B., Richert S., ... & Hore P. J. (2021). Magnetic sensitivity of cryptochrome 4 from a migratory songbird. Nature, 594(7864), 535–540. 34163056 10.1038/s41586-021-03618-9

[9] Dodson C. A., Hore P. J., & Wallace M. I. (2013). A radical sense of direction: signalling and mechanism in cryptochrome magnetoreception. Trends in biochemical sciences, 38(9), 435–446. 23938034 10.1016/j.tibs.2013.07.002

[10] Günther A., Einwich A., Sjulstok E., Feederle R., Bolte P., Koch K. W., ... & Mouritsen H. (2018). Double-cone localization and seasonal expression pattern suggest a role in magnetoreception for European robin cryptochrome 4. Current Biology, 28(2), 211–223. 29307554 10.1016/j.cub.2017.12.003

[11] Ritz T., Adem S., & Schulten K. (2000). A model for photoreceptor-based magnetoreception in birds. Biophysical journal, 78(2), 707–718. 10653784 10.1016/S0006-3495(00)76629-X PMC1300674

[12] Kao Y. T., Saxena C., He T. F., Guo L., Wang L., Sancar A., & Zhong D. (2008). Ultrafast dynamics of flavins in five redox states. Journal of the American Chemical Society, 130(39), 13132–13139. 18767842 10.1021/ja8045469 PMC2637805

[13] Liu B., Liu H., Zhong D., & Lin C. (2010). Searching for a photocycle of the cryptochrome photoreceptors. Current opinion in plant biology, 13(5), 578–586. 20943427 10.1016/j.pbi.2010.09.005 PMC2972227

[14] Schwinn K., Ferré N., & Huix-Rotllant M. (2020). UV-visible absorption spectrum of FAD and its reduced forms embedded in a cryptochrome protein. Physical Chemistry Chemical Physics, 22(22), 12447–12455. 32458897 10.1039/d0cp01714k

[15] Frederiksen A., Gerhards L., Reinholdt P., Kongsted J., & Solov’yov I. A. (2024). Importance of polarizable embedding for absorption spectrum calculations of arabidopsis thaliana cryptochrome 1. The Journal of Physical Chemistry B, 128(26), 6283–6290. 38913544 10.1021/acs.jpcb.4c02168 PMC11228989

[16] Nielsen C., Nørby M. S., Kongsted J., & Solov’yov I. A. (2018). Absorption spectra of FAD embedded in cryptochromes. The Journal of Physical Chemistry Letters, 9(13), 3618–3623. 29905481 10.1021/acs.jpclett.8b01528

[17] Kretschmer K., Frederiksen A., Reinholdt P., Kongsted J., & Solov’yov I. A. (2024). Understanding the red shift in the absorption spectrum of the FAD cofactor in CLCry4 protein. The Journal of Physical Chemistry B, 128(22), 5320–5326. 38805723 10.1021/acs.jpcb.4c00710 PMC11163422

[18] Van Der Spoel D., (2005). GROMACS: fast, flexible, and free. Journal of computational chemistry, 26(16), 1701–1718. 16211538 10.1002/jcc.20291

[19] Case D. A., (2005). The Amber biomolecular simulation programs. Journal of computational chemistry, 26(16), 1668–1688. 16200636 10.1002/jcc.20290 PMC1989667

[20] Vanommeslaeghe K., (2010). CHARMM general force field: A force field for drug-like molecules compatible with the CHARMM all-atom additive biological force fields. Journal of computational chemistry, 31(4), 671–690. 19575467 10.1002/jcc.21367 PMC2888302

[21] Dauber-Osguthorpe P., & Hagler A. T. (2019). Biomolecular force fields: where have we been, where are we now, where do we need to go and how do we get there? Journal of computer-aided molecular design, 33(2), 133–203. 30506158 10.1007/s10822-018-0111-4

[22] Soler J. M., (2002). The SIESTA method for ab initio order-N materials simulation. Journal of Physics: Condensed Matter, 14(11), 2745.

[23] Neese F. (2012). The ORCA program system. Wiley Interdisciplinary Reviews: Computational Molecular Science, 2(1), 73–78.

[24] Senn H. M., & Thiel W. (2009). QM/MM methods for biomolecular systems. Angewandte Chemie International Edition, 48(7), 1198–1229. 19173328 10.1002/anie.200802019

[25] Garcia M., & Wieners L. (in press). A quantum-mechanical framework for million-atom scale biological systems. Communications Chemistry. Preprint available at 10.21203/rs.3.rs-7327472/v1. PMC13128919 42056278

[26] Berman H. M., (2000). The protein data bank. Nucleic acids research, 28(1), 235–242. 10592235 10.1093/nar/28.1.235 PMC102472

[27] Liu Y., Zhao Z., Zhang X., Hao Y., Feng F., Chen Y., ... & Zhang P. (2025). Structural assembly of maize CRY-GL2 photosignaling complex provides insights into its regulatory role in cuticular wax biosynthesis. Science Advances, 11(49), eadz0136. 41337597 10.1126/sciadv.adz0136 PMC12674108

[28] Liu Y. & Zhang P., Crystal structure of maize CRY-GL2 photosignaling complex (2025) 10.2210/pdb9LPG/pdb PMC12674108 41337597

[29] Ma L., Wang X., Guan Z., Wang L., Wang Y., Zheng L. E., ... & Yin P. (2020). Structural insights into BIC-mediated inactivation of Arabidopsis cryptochrome 2. Nature Structural & Molecular Biology, 27(5), 472–479. 10.1038/s41594-020-0410-z 32398826

[30] Ma L., Wang X., Guan Z. & Yin P., Crystal structure of Arabidopsis thaliana CRY2 (2019) 10.2210/pdb6K8I/pdb

[31] Wang X., Ma L., Guan Z. & Yin P., Crystal structure of Arabidopsis thaliana BIC2-CRY2 complex (2019) 10.2210/pdb6K8K/pdb

[32] Palayam M., Ganapathy J., Guercio A. M., Tal L., Deck S. L., & Shabek N. (2021). Structural insights into photoactivation of plant Cryptochrome-2. Communications Biology, 4(1), 28. 33398020 10.1038/s42003-020-01531-x PMC7782693

[33] Palayam M., Ganapathy J., Guercio M.A. & Shabek N., Structural basis of plant blue light photoreceptor (2020) 10.2210/pdb6X24/pdb

[34] Zoltowski B. D., Chelliah Y., Wickramaratne A., Jarocha L., Karki N., Xu W., ... & Takahashi J. S. (2019). Chemical and structural analysis of a photoactive vertebrate cryptochrome from pigeon. Proceedings of the National Academy of Sciences, 116(39), 19449–19457. 10.1073/pnas.1907875116 PMC6765304 31484780

[35] Zoltowski B.D., Chelliah Y., Wickramaratne A.C., Jarocha L., Karki N., Mouritsen H., Hore P.J., Hibbs R.E., Green C.B. & Takahashi J.S., Pigeon Cryptochrome4 bound to flavin adenine dinucleotide (2019) 10.2210/pdb6PU0/pdb PMC6765304 31484780

[36] Chandrasekaran S., Schneps C. M., Dunleavy R., Lin C., DeOliveira C. C., Ganguly A., & Crane B. R. (2021). Tuning flavin environment to detect and control light-induced conformational switching in Drosophila cryptochrome. Communications biology, 4(1), 249. 33637846 10.1038/s42003-021-01766-2 PMC7910608

[37] Schneps C.M. & Crane B.R., Sort-Tagged Drosophila Cryptochrome (2020) 10.2210/pdb6WTB/pdb

[38] Vu H. H., Behrmann H., Hanić M., Jeyasankar G., Krishnan S., Dannecker D., ... & Behrmann E. (2023). A marine cryptochrome with an inverse photo-oligomerization mechanism. Nature Communications, 14(1), 6918. 10.1038/s41467-023-42708-2 PMC10616196 37903809

[39] Behrmann E. & Behrmann H., FAD_ox bound dark state structure of PdLCry (2023) 10.2210/pdb8P4X/pdb

[40] Müller C., Sharma M., & Sierka M. (2020). Real-time time-dependent density functional theory using density fitting and the continuous fast multipole method. Journal of Computational Chemistry, 41(30), 2573–2582. 33464600 10.1002/jcc.26412

[41] Jacquemin D., Perpete E. A., Scuseria G. E., Ciofini I., & Adamo C. (2008). TD-DFT performance for the visible absorption spectra of organic dyes: conventional versus long-range hybrids. Journal of chemical theory and computation, 4(1), 123–135. 26619986 10.1021/ct700187z

[42] Wu X., Xie X., & Troisi A. (2024). Calibration of several first excited state properties for organic molecules through systematic comparison of TDDFT with experimental spectra. Journal of Materials Chemistry C, 12(46), 18886–18892. 39444434 10.1039/d4tc03511a PMC11492815

[43] Lewis J. A., & Escalante-Semerena J. C. (2006). The FAD-dependent tricarballylate dehydrogenase (TcuA) enzyme of Salmonella enterica converts tricarballylate into cis-aconitate. Journal of bacteriology, 188(15), 5479–5486. 16855237 10.1128/JB.00514-06 PMC1540016

[44] Müller P., Ignatz E., Kiontke S., Brettel K., & Essen L. O. (2018). Sub-nanosecond tryptophan radical deprotonation mediated by a protein-bound water cluster in class II DNA photolyases. Chemical science, 9(5), 1200–1212. 29675165 10.1039/c7sc03969g PMC5885780

[45] Li X., Wang Q., Yu X., Liu H., Yang H., Zhao C., ... & Lin C. (2011). Arabidopsis cryptochrome 2 (CRY2) functions by the photoactivation mechanism distinct from the tryptophan (trp) triad-dependent photoreduction. Proceedings of the National Academy of Sciences, 108(51), 20844–20849. 10.1073/pnas.1114579108 PMC3251054 22139370

[46] Lin C., Top D., Manahan C. C., Young M. W., & Crane B. R. (2018). Circadian clock activity of cryptochrome relies on tryptophan-mediated photoreduction. Proceedings of the National Academy of Sciences, 115(15), 3822–3827. 10.1073/pnas.1719376115 PMC5899454 29581265

[47] Levy C., Zoltowski B. D., Jones A. R., Vaidya A. T., Top D., Widom J., ... & Leys D. (2013). Updated structure of Drosophila cryptochrome. Nature, 495(7441), E3–E4. 23518567 10.1038/nature11995 PMC3694752

[48] Zoltowski B.D., Vaidya A.T., Top D., Widom J., Young M.W., Levy C., Jones A.R., Scrutton N.S., Leys D. & Crane B.R., Structure of Full-length Drosophila Cryptochrome (2012) 10.2210/pdb4GU5/pdb PMC3240699 22080955

[49] Klar T., Pokorny R., Moldt J., Batschauer A., & Essen L. O. (2007). Cryptochrome 3 from Arabidopsis thaliana: structural and functional analysis of its complex with a folate light antenna. Journal of molecular biology, 366(3), 954–964. 17188299 10.1016/j.jmb.2006.11.066

[50] Klar T., Pokorny R., Batschauer A. & Essen L.-O., Cryptochrome 3 from Arabidopsis thaliana (2006) 10.2210/pdb2J4D/pdb PMC1991327 16511200

[51] Garcia M., & Wieners L. (2025). Linking UV-induced DNA damage with base pair sequences. [Manuscript in preparation]

[52] Meng E. C., (2023). UCSF ChimeraX: Tools for structure building and analysis. Protein Science, 32(11), e4792. 37774136 10.1002/pro.4792 PMC10588335

[53] Pettersen E. F., UCSF ChimeraX: Structure visualization for researchers, educators, and developers. Protein science, 30(1), 70–82. 10.1002/pro.3943 PMC7737788 32881101

[54] Goddard T. D., (2018). UCSF ChimeraX: Meeting modern challenges in visualization and analysis. Protein science, 27(1), 14–25. 28710774 10.1002/pro.3235 PMC5734306

[55] Huang Y., Baxter R., Smith B. S., Partch C. L., Colbert C. L., & Deisenhofer J. (2006). Crystal structure of cryptochrome 3 from Arabidopsis thaliana and its implications for photolyase activity. Proceedings of the National Academy of Sciences, 103(47), 17701–17706. 10.1073/pnas.0608554103 PMC1635974 17101984

[56] Juhas M., von Zadow A., Spexard M., Schmidt M., Kottke T., & Büchel C. (2014). A novel cryptochrome in the diatom P haeodactylum tricornutum influences the regulation of light-harvesting protein levels. The FEBS journal, 281(9), 2299–2311. 24628952 10.1111/febs.12782

[57] Müller P., Yamamoto J., Martin R., Iwai S., & Brettel K. (2015). Discovery and functional analysis of a 4th electron-transferring tryptophan conserved exclusively in animal cryptochromes and (6–4) photolyases. Chemical Communications, 51(85), 15502–15505. 26355419 10.1039/c5cc06276d

[58] Cailliez F., Müller P., Firmino T., Pernot P., & de La Lande A. (2016). Energetics of photoinduced charge migration within the tryptophan tetrad of an animal (6–4) photolyase. Journal of the American Chemical Society, 138(6), 1904–1915. 26765169 10.1021/jacs.5b10938

[59] Pritchard B. P., Altarawy D., Didier B., Gibson T. D., & Windus T. L. (2019). New basis set exchange: An open, up-to-date resource for the molecular sciences community. Journal of chemical information and modeling, 59(11), 4814–4820. 31600445 10.1021/acs.jcim.9b00725

[60] Paszke A. (2019). Pytorch: An imperative style, high-performance deep learning library. arXiv preprint arXiv:1912.01703.

[61] Lam S. K., Pitrou A., & Seibert S. (2015, November). Numba: A llvm-based python jit compiler. In: Proceedings of the Second Workshop on the LLVM Compiler Infrastructure in HPC (pp. 1–6).

[62] Hunter J. D. (2007). Matplotlib: A 2D graphics environment. Computing in science & engineering, 9(03), 90–95.

[63] Andermatt S., Cha J., Schiffmann F., & VandeVondele J. (2016). Combining linear-scaling DFT with subsystem DFT in Born–Oppenheimer and Ehrenfest molecular dynamics simulations: From molecules to a virus in solution. Journal of chemical theory and computation, 12(7), 3214–3227. 27244103 10.1021/acs.jctc.6b00398

[64] Shao K., Zhang X., Li X., Hao Y., Huang X., Ma M., ... & Zhang P. (2020). The oligomeric structures of plant cryptochromes. Nature Structural & Molecular Biology, 27(5), 480–488. 10.1038/s41594-020-0420-x 32398825

[65] Pokorny R., Klar T., Hennecke U., Carell T., Batschauer A., & Essen L. O. (2008). Recognition and repair of UV lesions in loop structures of duplex DNA by DASH-type cryptochrome. Proceedings of the National Academy of Sciences, 105(52), 21023–21027. 10.1073/pnas.0805830106 PMC2634942 19074258

[66] Cakilkaya B., Kavakli I. H., & DeMirci H. (2023). The crystal structure of Vibrio cholerae (6–4) photolyase reveals interactions with cofactors and a DNA-binding region. Journal of Biological Chemistry, 299(1), 102794. 36528063 10.1016/j.jbc.2022.102794 PMC9852545

[67] Emmerich H. J., Schneider L., & Essen L. O. (2023). Structural and Functional Analysis of a Prokaryotic (6–4) Photolyase from the Aquatic Pathogen Vibrio Cholerae. Photochemistry and Photobiology, 99(5), 1248–1257. 36692077 10.1111/php.13783

[68] Wang P. H., Hosokawa Y., C Soares J., Emmerich H. J., Fuchs V., Caramello N., ... & Essen L. O. (2025). Redox-State-Dependent Structural Changes within a Prokaryotic 6–4 Photolyase. Journal of the American Chemical Society, 147(19), 16084–16098. 40298610 10.1021/jacs.4c18116 PMC12082625

[69] Brudler R., Hitomi K., Daiyasu H., Toh H., Kucho K. I., Ishiura M., ... & Getzoff E. D. (2003). Identification of a new cryptochrome class: structure, function, and evolution. Molecular cell, 11(1), 59–67. 12535521 10.1016/s1097-2765(03)00008-x

[70] Xing W., Busino L., Hinds T. R., Marionni S. T., Saifee N. H., Bush M. F., ... & Zheng N. (2013). SCFFBXL3 ubiquitin ligase targets cryptochromes at their cofactor pocket. Nature, 496(7443), 64–68. 23503662 10.1038/nature11964 PMC3618506

[71] Ma L., Guan Z., Wang Q., Yan X., Wang J., Wang Z., ... & Yin P. (2020). Structural insights into the photoactivation of Arabidopsis CRY2. Nature plants, 6(12), 1432–1438. 33199893 10.1038/s41477-020-00800-1

[72] Hao Y., Zhang X., Liu Y., Ma M., Huang X., Liu H., & Zhang P. (2023). Cryo–EM structure of the CRY2 and CIB1 fragment complex provides insights into CIB1-mediated photosignaling. Plant communications, 4(2). 10.1016/j.xplc.2022.100475 PMC10030363 36371635

[73] Brautigam C. A., Smith B. S., Ma Z., Palnitkar M., Tomchick D. R., Machius M., & Deisenhofer J. (2004). Structure of the photolyase-like domain of cryptochrome 1 from Arabidopsis thaliana. Proceedings of the National Academy of Sciences, 101(33), 12142–12147. 10.1073/pnas.0404851101 PMC514401 15299148

[74] Czarna A., Berndt A., Singh H. R., Grudziecki A., Ladurner A. G., Timinszky G., ... & Wolf E. (2013). Structures of Drosophila cryptochrome and mouse cryptochrome1 provide insight into circadian function. Cell, 153(6), 1394–1405. 23746849 10.1016/j.cell.2013.05.011

[75] Schneps C. M., Ganguly A., & Crane B. R. (2022). Room-temperature serial synchrotron crystallography of Drosophila cryptochrome. Biological Crystallography, 78(8), 975–985. 10.1107/S2059798322007008 PMC9344480 35916222

[76] Lin C., Feng S., DeOliveira C. C., & Crane B. R. (2023). Cryptochrome–Timeless structure reveals circadian clock timing mechanisms. Nature, 617(7959), 194–199. 37100907 10.1038/s41586-023-06009-4 PMC11034853

[77] Shao K., Zhang X. & Zhang P., Structure of cryptochrome in active conformation (2020) 10.2210/pdb6LZ3/pdb

[78] Huang Y. & Deisenhofer J., Crystal Structure of cryptochrome 3 from Arabidopsis thaliana (2006) 10.2210/pdb2IJG/pdb PMC1635974 17101984

[79] Pokorny R., Klar T., Hennecke U., Carell T., Batschauer A. & Essen L.-O., Structure of cryptochrome 3 - DNA complex (2008) 10.2210/pdb2VTB/pdb PMC2634942 19074258

[80] Cakilkaya B., Kavakli I.H. & DeMirci H., Crystal structure of (6–4) photolyase from Vibrio cholerae (2022) 10.2210/pdb7YKN/pdb PMC9852545 36528063

[81] Essen L.-O. & Emmerich H.J., Bacterial 6–4 photolyase from Vibrio cholerase (2022) 10.2210/pdb8A1H/pdb

[82] Po Hsun W., Maestre-Reyna M. & Essen L.-O., Structure of the (6–4) photolyase of Caulobacter crescentus in its oxidizd state - Singal crystal / Synchrotron (2024) 10.2210/pdb9HNK/pdb

[83] Po Hsun W., Maestre-Reyna M. & Essen L.-O., Structure of the (6–4) photolyase of Caulobacter crescentus in its oxidized state at room temperture-synchrotron (2024) 10.2210/pdb9HNL/pdb

[84] Po Hsun W., Maestre-Reyna M. & Essen L.-O., Structure of the (6–4) photolyase of Caulobacter crescentus in its dark adapted and oxidized state determined by serial femtosecond crystallography (2024) 10.2210/pdb9HNM/pdb

[85] Po Hsun W., Maestre-Reyna M. & Essen L.-O., Structure of the (6–4) photolyase of Caulobacter crescentus in its fully reduced state determined by serial femtosecond crystallography (2024) 10.2210/pdb9HNN/pdb

[86] Po Hsun W., Maestre-Reyna M. & Essen L.-O., Structure of the (6–4) photolyase of Caulobacter crescentus in its iron sulfur cluster oxidized state determined by serial femtosecond crystallography (2024) 10.2210/pdb9HNO/pdb

[87] Po Hsun W., Maestre-Reyna M. & Essen L.-O., Structure of the (6–4) photolyase of Caulobacter crescentus with K48A mutation in its dark adapted and oxidized state determined by synchrotron (2025) 10.2210/pdb9Q8F/pdb

[88] Brudler R., Hitomi K., Daiyasu H., Toh H., Kucho K., Ishiura M., Kanehisa M., Roberts V.A., Todo T., Tainer J.A. & Getzoff E.D., Crystal Structure Analysis of Synechocystis sp. PCC6803 cryptochrome (2003) 10.2210/pdb1NP7/pdb

[89] Xing W., Busino L., Hinds T.R., Marionni S.T., Saifee N.H., Bush M.F., Pagano M. & Zheng N., a vertebrate cryptochrome with FAD (2012) 10.2210/pdb4I6G/pdb PMC3618506 23503662

[90] Ma L., Guan Z.Y. & Yin P., Cryo-EM structure of Arabidopsis CRY under blue light-mediated activation (2020) 10.2210/pdb6M79/pdb

[91] Hao Y.H., Zhang X. & Zhang P., Cryo-EM Structure of Arabidopsis CRY2 in active conformation (2022) 10.2210/pdb7X0X/pdb

[92] Hao Y.H., Zhang X. & Zhang P., Cryo-EM Structure of Arabidopsis CRY2 tetramer in complex with CIB1 fragment (2022) 10.2210/pdb7X0Y/pdb

[93] Brautigam C.A., Smith B.S., Ma Z., Palnitkar M., Tomchick D.R., Machius M. & Deisenhofer J., Crystal Structure of the PHR domain of Cryptochrome 1 from Arabidopsis thaliana (2004) 10.2210/pdb1U3C/pdb PMC514401 15299148

[94] Brautigam C.A., Smith B.S., Ma Z., Palnitkar M., Tomchick D.R., Machius M. & Deisenhofer J., Crystal Structure of the PHR domain of Cryptochrome 1 from Arabidopsis thaliana with AMPPNP bound (2004) 10.2210/pdb1U3D/pdb PMC514401 15299148

[95] Czarna A. & Wolf E., Crystal structures of Drosophila Cryptochrome (2013) 10.2210/pdb4JZY/pdb 23746849

[96] Berndt A. & Wolf E., Crystal structure of Drosophila Cryprochrome (2013) 10.2210/pdb4K03/pdb

[97] Shao K., Zhang X. & Zhang P., Tetrameric structure of ZmCRY1a PHR domain (2020) 10.2210/pdb6LZ7/pdb

[98] Zoltowski B.D., Chelliah Y., Wickramaratne A.C., Jarocha L., Karki N., Mouritsen H., Hore P.J., Hibbs R.E., Green C.B. & Takahashi J.S., Crystal structure of pigeon Cryptochrome 4 mutant Y319D in complex with flavin adenine dinucleotide (2019) 10.2210/pdb6PTZ/pdb PMC6765304 31484780

[99] Schneps C.M. & Crane B.R., Room Temperature Drosophila Cryptochrome (2022) 10.2210/pdb7UD0/pdb PMC9344480 35916222

[100] Feng S., Lin C., DeOliveira C.C. & Crane B.R., The Cryo-EM structure of Drosophila Cryptochrome in complex with Timeless (2022) 10.2210/pdb8DD7/pdb

